# The Arginine Repressor ArgR_2_ Controls Conjugated Linoleic Acid Biosynthesis by Activating the *cla* Operon in *Lactiplantibacillus plantarum*

**DOI:** 10.1128/spectrum.02619-21

**Published:** 2022-06-02

**Authors:** Xin-Xin Liu, Lei Liu, Xin Song, Guang-Qiang Wang, Zhi-Qiang Xiong, Yong-Jun Xia, Lian-Zhong Ai

**Affiliations:** a Shanghai Engineering Research Center of Food Microbiology, School of Health Science and Engineering, University of Shanghai for Science and Technology, Shanghai, China; Francis Crick Institute

**Keywords:** CLA, conjugated linoleic acid, *Lactiplantibacillus plantarum*, biosynthesis, ArgR_2_, arginine repressor, transcriptional regulation

## Abstract

CLA (conjugated linoleic acid) has attracted substantial attention due to its physiological functions, including regulating immunity, reducing obesity, and contributing to cancer suppression. In Lactiplantibacillus plantarum, CLA oleate hydratase (CLA-HY), CLA short-chain dehydrogenase (CLA-DH), and CLA acetoacetate decarboxylase (CLA-DC) catalyze the biotransformation of linoleic acid (LA) to CLA. However, the underlying transcriptional regulation mechanism of this pathway remains largely unknown. In this study, the potential transcriptional regulators that might bind to the *cla* promoter of L. plantarum AR195 were investigated by DNA pulldown. Interestingly, ArgR_2_, the transcriptional regulator of arginine metabolism, was identified as a potential regulator involved in the regulation of CLA biotransformation. Electrophoretic mobility shift assay (EMSA) and molecular interaction results demonstrated the specific binding of ArgR_2_ to the regulatory region of the *cla* operon. The knockout of *argR_2_* led to the downregulation of *cla-dh* and *cla-dc* by 91% and 34%, respectively, resulting in a decline in the CLA yield by 14%. A segmental EMSA revealed that ArgR_2_ bound to three distinct sites in the *cla* regulatory region, and these binding sites were highly conserved and rich in AT. The regulatory mechanism of ArgR_2_ on CLA biosynthesis further expanded our knowledge of the regulatory mechanism of CLA biosynthesis in *L. plantarum* and laid the theoretical foundation for the production and application of CLA.

**IMPORTANCE** CLA (conjugated linoleic acid) has received extensive attention owing to its important physiological functions. CLA from natural sources is far from meeting people’s demands. Lactic acid bacteria can efficiently synthesize *cis*-9,*trans*-11-CLA and *trans*-10,*cis*-12-CLA, which possess physiological activities. However, little is known about the regulatory mechanism. In this study, we identified that the biosynthesis of CLA in *L. plantarum* AR195 was transcriptionally regulated by the arginine biosynthesis regulatory protein ArgR_2_. The regulation mechanism of ArgR_2_ on CLA biosynthesis lays a theoretical foundation for the regulation of CLA synthesis and industrial production.

## INTRODUCTION

CLA (conjugated linoleic acid) is the general term for a variety of octadecadienoic acid isomers containing conjugated double bonds. CLA has gained much attention for its various health-related physiological activities, including its anticancer properties, antiatherosclerosis properties, inhibition of inflammation, inhibition of obesity, inhibition of diabetes, and prevention of cardiovascular diseases (1–3). There has been sufficient evidence to prove that the physiological functions of CLA are attributed mainly to two isomers: *cis*-9,*trans*-11-CLA (*c*9,*t*11-CLA) and *trans*-10,*cis*-12-CLA (1, 4).

Rumen dairy products and meat products are the main sources of CLA in people’s daily life. However, the content of CLA in these products is too low to have a physiological function. Moreover, there is a lack of research on its biosynthesis and regulation mechanisms. The typical microorganisms with CLA biosynthesis capabilities include rumen bacteria, *Propionibacterium*, and *Lactobacillus* (1). Recently, an increasing number of studies have focused on CLA biosynthesis by *Lactobacillus*. The main reasons include the following. First, compared with rumen bacteria and *Propionibacterium*, *Lactobacillus* has the advantages of being safe and amenable to easy large-scale culture (5). Functional foods including CLA produced by *Lactobacillus* are more acceptable to people. Second, *Lactobacillus* can synthesize *c*9,*t*11-CLA and *t*10,*c*12-CLA with higher isomer selectivity. The CLA synthesized by *Lactobacillus* has broad application prospects in the food industry due to its specific functions.

The lactobacilli with CLA biotransformation abilities cover almost all species and genera of lactic acid bacteria, including Lactiplantibacillus plantarum (6–8), Lactobacillus acidophilus (9), Lactobacillus pentosus (10), Lactobacillus reuteri (11), Lactococcus lactis (12), and *Bifidobacterium* (13), etc. CLA isomerase is considered the key enzyme in CLA biosynthesis. Rosson et al. heterologously expressed CLA isomerase of Lactobacillus reuteri ATCC 55739. However, no CLA was detected (14). Furthermore, hydroxyl fatty acid was identified as the intermediate product during CLA bioconversion in L. plantarum AKU 1137, demonstrating that the biotransformation of CLA from linoleic acid (LA) in *Lactobacillus* was not a one-step isomerization (15). After that, Kishino et al. identified the genes encoding the enzymes involved in CLA biotransformation in *L. plantarum* AKU 1009a and revealed the synthetic pathway in detail (16).

In *L. plantarum*, linoleic acid was first hydrated to 10-hydroxy-cis12-octadecenoic acid (10-HOE) under the catalysis of CLA oleate hydratase (CLA-HY) (hydration/dehydration). After dehydration, double-bond shift, hydration, and dehydration reactions, 10-HOE was finally transformed into CLA. The enzymes involved in this process included CLA-HY, CLA short-chain dehydrogenase (CLA-DH), and CLA acetoacetate decarboxylase (CLA-DC) (16). Yang et al. also verified this pathway in *L. plantarum* ZS2058 based on *cre-lox* gene-editing technology (17, 18). Moreover, our previous work had proven that strains with different CLA biosynthesis abilities possessed different transcriptional levels of *cla-hy*, *cla-dh*, and *cla-dc*, suggesting that the upregulation of the CLA yield may be achieved by regulating the transcription of these genes (19). Except for *Lp*LttR, the LysR-type transcriptional regulator of *L. plantarum* WCFS1 (20), we speculated that there may be other regulators involved in CLA synthesis.

In this study, we found that ArgR_2_ bound to the regulatory region of the *cla* operon in *L. plantarum* AR195. The knockout of *argR_2_* showed no obvious effect on bacterial growth, but it showed an inhibitory effect on CLA biosynthesis. The regulation of CLA biotransformation by ArgR_2_ was achieved by binding to three different sites on the regulatory region of the *cla* operon. This study broadened the understanding of ArgR_2_ in *L. plantarum* and provided a theoretical basis for the regulation and production of CLA.

## RESULTS

### ArgR_2_ was the potential regulator of CLA biosynthesis.

**FIG 1:**
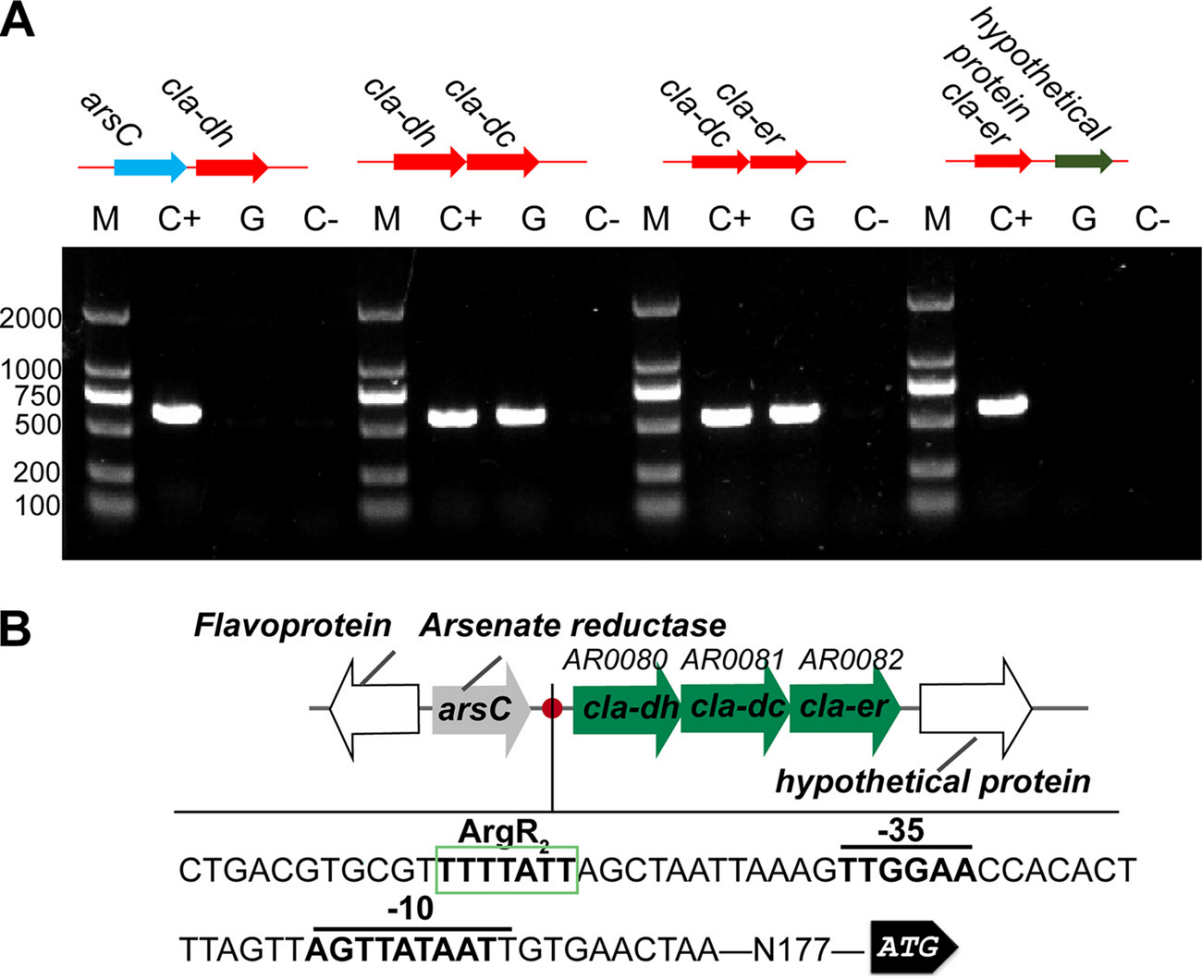
Gene structure and *cis*-element analysis of the *cla* operon. (A) Analysis of the transcriptional organization of *cla* clusters in *L. plantarum* AR195. M, DNA marker; C+, positive control using the genomic DNA as the template; G, fragment PCR using the cDNA as the template; C−, negative control using the total RNA as the template. (B) *cis*-element analysis of the *cla* promoter in *L. plantarum* AR195. *cla-dh*, *cla-dc*, and *cla-er*, highlighted in green, are located in the same transcriptional unit.

In our previous study, *L. plantarum* AR195 showed the highest CLA biotransformation capability among the *L. plantarum* strains in our laboratory stock (19). To investigate the biosynthesis and regulation mechanisms of CLA in *L. plantarum* AR195, the detailed genome was sequenced. The genome size was 5.28 Mb, and it also contained multiple plasmids. The genome length is 3,219,240 bp, and 3,235 coding genes were predicted. The genes encoding CLA-HY, CLA-DH, and CLA-DC in *L. plantarum* AR195 are *AR0148*, *AR0080*, and *AR0081*, respectively. Amplification of the overlapping regions was performed to investigate the gene structure and verify the promoter. RNA and cDNA were used as the templates for PCR amplification, respectively, and the genomic DNA was used as a control. As shown in Fig. 1A, *cla-dh* and *cla-dc* were located in one transcriptional unit, which was similar to that in *L. plantarum* WCFS1. Moreover, *cla-er* (*AR0082*) was also located in this operon.

To explore the potential transcriptional regulators that might regulate CLA biotransformation in *L. plantarum* AR195, a DNA pulldown experiment was carried out. Interestingly, an arginine metabolism repressor, ArgR_2_, was captured, indicating that ArgR_2_ was most likely the potential regulator of CLA biosynthesis. *cis*-element analysis of the *cla* operon was also performed using the Softberry website (http://linux1.softberry.com/berry.phtml?topic=bprom&group=programs&subgroup=gfindb). A potential ArgR binding box was found in the *cla* promoter at a distance of 12 bp from the −35 region (Fig. 1B). According to previous reports, ArgR_2_ mainly regulated the expression of genes related to arginine metabolism, glutamic acid metabolism, lysine metabolism, purine and pyrimidine synthesis, and cell morphology. It has not been reported that it participated in fatty acid metabolism, especially the biosynthesis of CLA. To investigate the function of ArgR_2_, *argR_2_* heterologous expression and knockout mutant strains were constructed.

### Heterologous expression of ArgR_2_.

**FIG 2:**
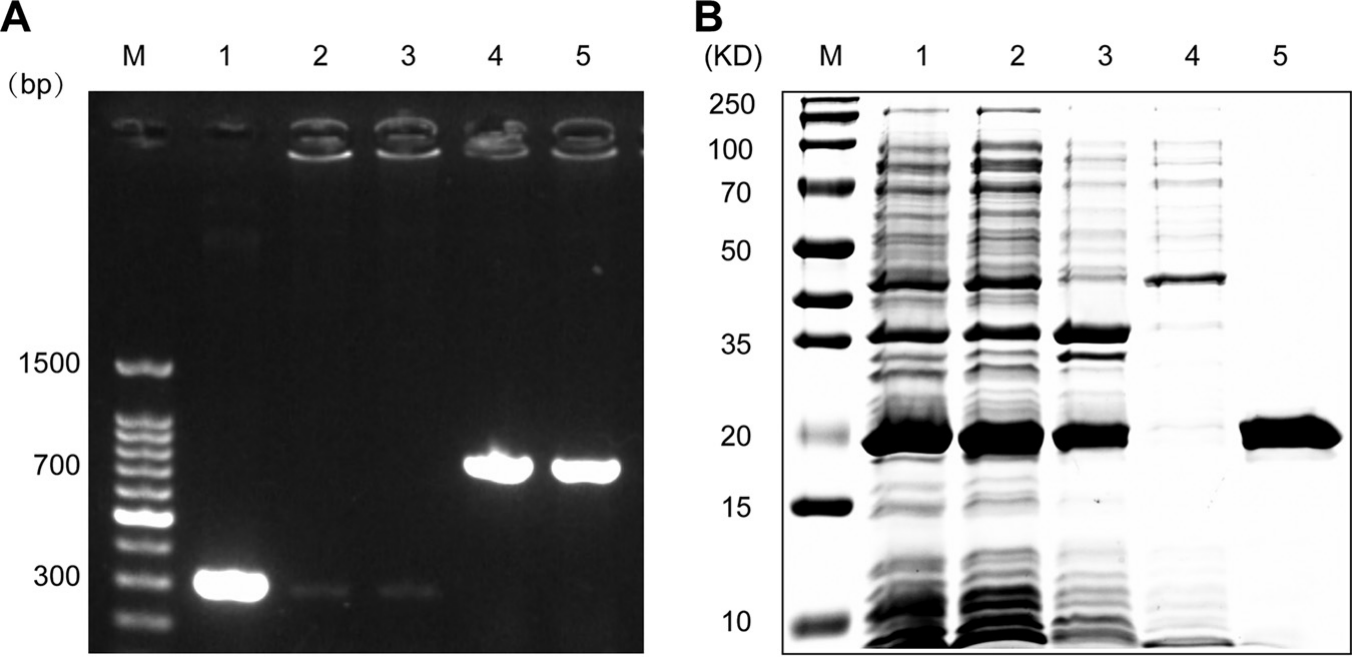
Construction and heterologous expression of ArgR_2_. (A) Agarose gel electrophoresis of colony PCR products. Lanes 1 to 5 show colony PCR products of 5 selected colonies. (B) SDS-PAGE of His-ArgR_2_. Lane 1, component after ultrasonic crushing; lane 2, the centrifugal supernatant; lane 3, the precipitate; lane 4, eluted miscellaneous protein; lane 5, purified His-ArgR_2_.

ArgR_2_ of *L. plantarum* AR195 contains 459 bases, encoding 152 amino acids. The molecular weight was 17.5 kDa, predicted by the amino acid sequence using ExPASY (https://web.expasy.org/compute_pi/). The positive clones were screened by colony PCR and sequence verification (Fig. 2A). The positive colonies (Fig. 2A, lane 4) were used for protein expression. As shown in Fig. 2B, ArgR_2_ was successfully expressed as a soluble product. The molecular weight of ArgR_2_ matched well with the theoretical molecular weight, and protein purification was successful, without further contamination.

### ArgR_2_ bound to the promoter of the *cla* operon.

**FIG 3:**
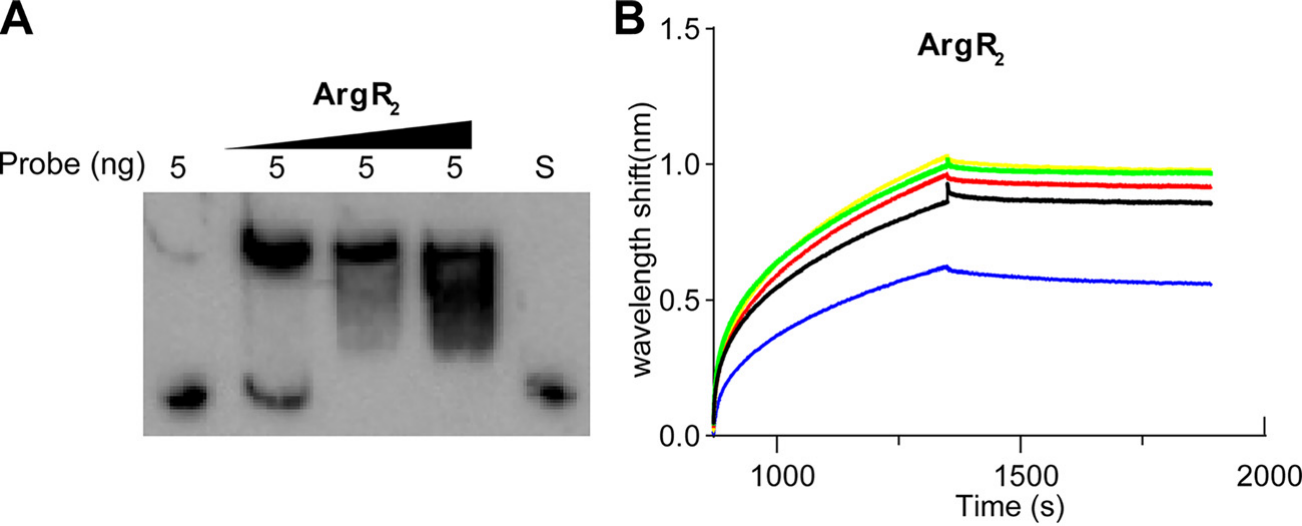
Protein ArgR_2_ and DNA interaction. (A) EMSA of ArgR_2_ binding with the *cla* promoter. S represents the negative control using the unlabeled probe. Lanes 1 to 4 show ArgR_2_ at 0, 0.0425, 0.085, and 0.17 mg/mL. (B) Kinetic binding of ArgR_2_ and DNA analyzed by Octet biolayer interferometry. ArgR_2_ concentrations are 4,097, 2,049, 1,024, 512, and 256 nM.

To investigate whether ArgR_2_ bound to the regulatory region of the *cla* operon *in vitro*, electrophoretic mobility shift assays (EMSAs) and Octet experiments were performed. As shown in Fig. 3A, a DNA-protein complex band was detected when ArgR_2_ was incubated with the biotin-labeled probes. Moreover, the binding band showed a concentration-dependent increase. When the protein reached 0.085 mg/mL, binding was saturated. Binding was further verified by an Octet experiment. The ArgR_2_-*cla* promoter interaction showed that the affinity coefficient (*K_D_*) was less than 1.0 pM (Fig. 3B). These results indicated that ArgR_2_ stably bound to the *cla* promoter, suggesting that ArgR_2_ might be involved in the regulation of CLA biotransformation.

### Construction of the *argR_2_* knockout strain.

**FIG 4:**
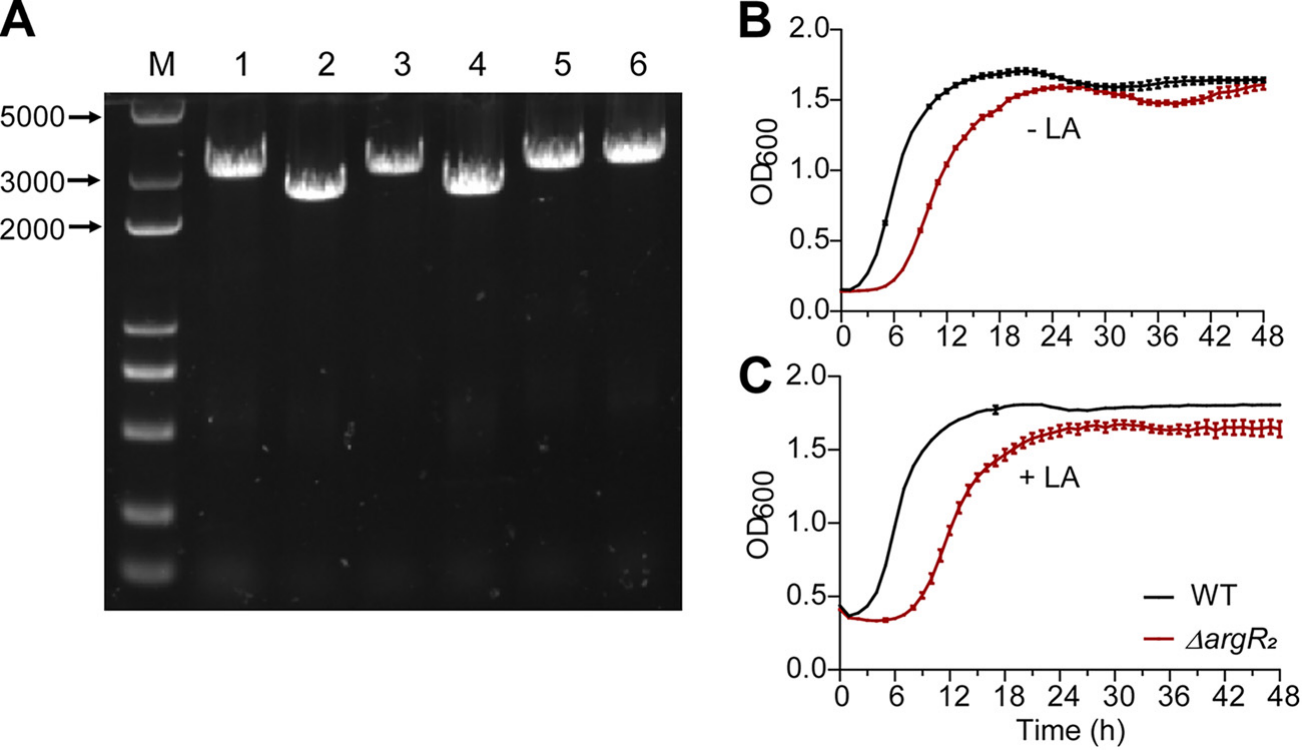
Construction of the *argR_2_* knockout strain. (A) PCR verification of the selected colonies. Lane 1, wild-type strain; lanes 2 to 6, PCR verification of the selected colonies. (B) Bacterial growth of WT and Δ*argR_2_* strains cultured in MRS medium without LA. (C) Bacterial growth of WT and Δ*argR_2_* strains cultured in MRS medium with LA. Error bars show standard deviations from three independent experiments. OD_600_, optical density at 600 nm.

The *argR_2_* knockout strain was necessary to investigate the regulatory effect of ArgR_2_ on CLA biotransformation. Therefore, we constructed an *argR_2_* knockout strain of *L. plantarum* AR195, Δ*argR_2_*, using the CRISPR-Cas9 system (Fig. 4A). According to previous studies, ArgR_2_ is involved in nitrogen metabolism, purine synthesis, pyrimidine synthesis, cell morphology, and so on. The effect of the *argR_2_* mutation on the growth of *L. plantarum* AR195 was investigated in this study. As shown in Fig. 4B, the deletion of *argR_2_* slowed bacterial growth in the first 24 h, but the final biomass was close to that when bacteria were cultured in MRS broth without LA. However, when the medium was supplemented with LA, the growth ability of the knockout strain (Δ*argR_2_*) was suppressed to a modest degree, indicating that ArgR_2_ played a role in the biotransformation of CLA from LA.

### ArgR_2_ controls CLA biotransformation by regulating the transcription of the *cla* operon.

**FIG 5:**
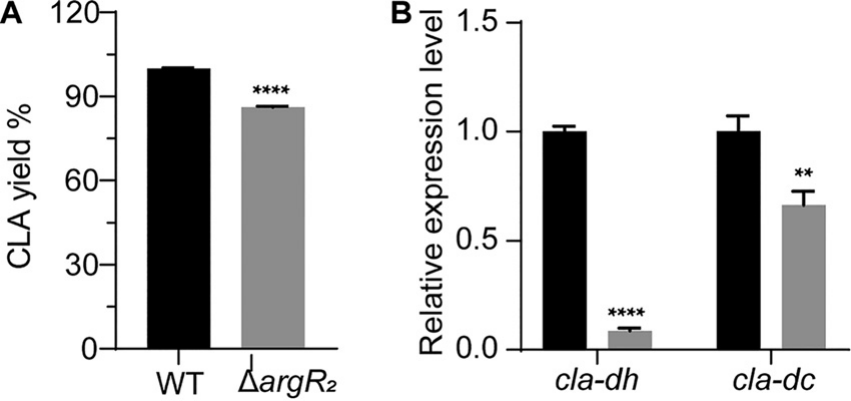
ArgR_2_ transcriptionally activated CLA biotransformation. (A) CLA concentrations in the WT and Δ*argR_2_* strains. (B) Real-time fluorescence quantitative PCR analysis of the CLA-related genes. Error bars show standard deviations from three independent experiments. *P* values were determined by the two-tailed unpaired *t* test. *, *P* < 0.05; **, *P* < 0.01; ***, *P* < 0.001; ****, *P* < 0.0001.

To investigate the effect of ArgR_2_ on CLA biosynthesis, the levels of CLA production in the wild-type (WT) and Δ*argR_2_* strains were determined. As shown in Fig. 5A, the knockout of *argR_2_* decreased CLA production from 107.3 to 92.4 μg/mL, reducing the CLA yield by 14%. We speculated that the inhibitory effect of *argR_2_* on the CLA yield might be achieved by regulating the expression of CLA synthesis genes. To test this hypothesis, the transcriptional levels of CLA synthesis genes in the WT and Δ*argR_2_* strains were examined. RNA was isolated when the cells were grown to the mid-exponential phase. Compared with those in the WT, the knockout of *argR_2_* decreased the transcription levels of *cla-dh* and *cla-dc* by 91% and 34%, respectively (Fig. 5B), suggesting that ArgR_2_ regulated the biosynthesis of CLA by positively regulating the transcription of *cla-dh* and *cla-dc*.

### Identification of the regulatory site.

**FIG 6:**
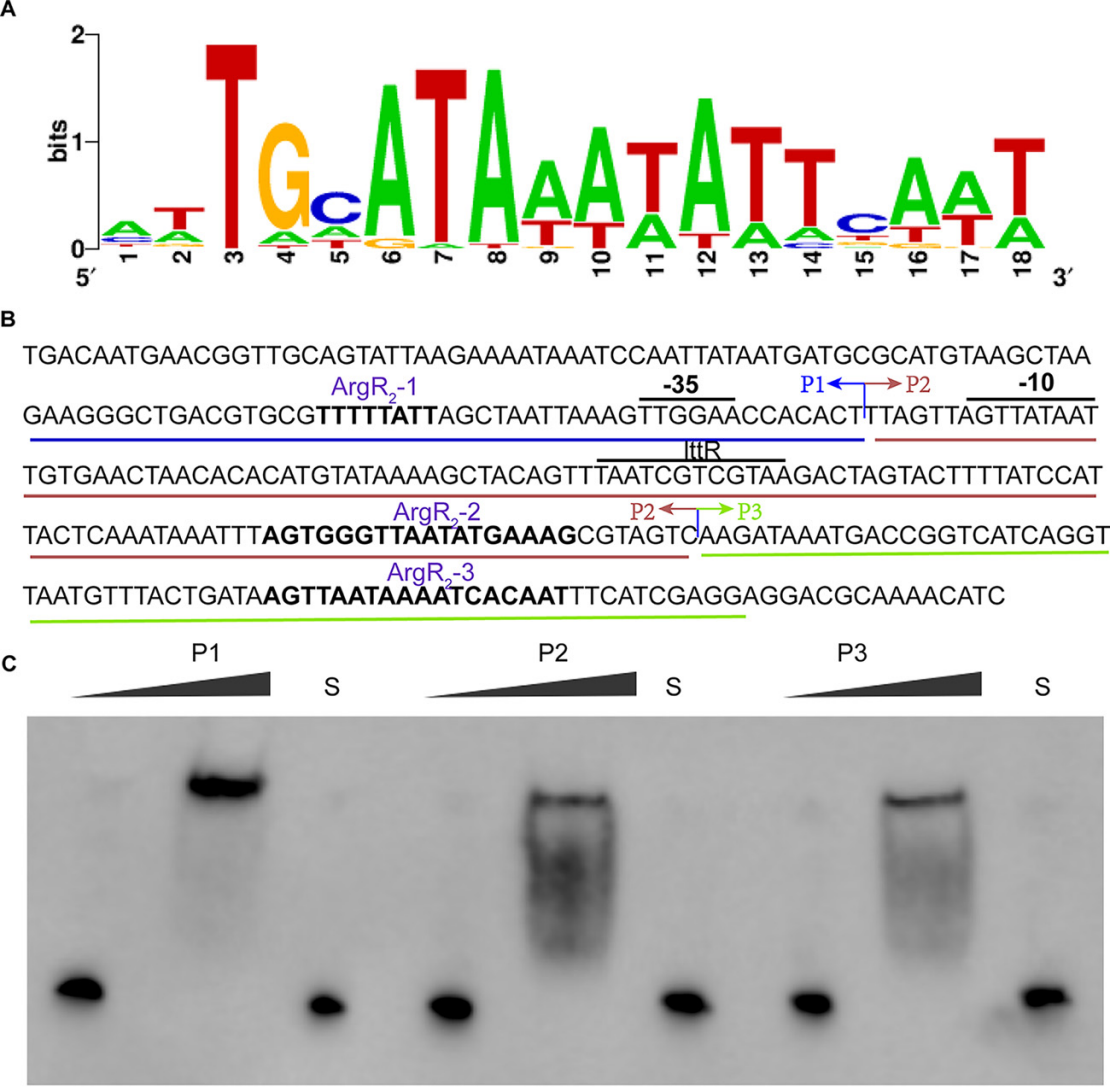
Identification of the binding site of ArgR_2_. (A) ArgR binding motif predicted by regprecise. (B) Putative binding sites of ArgR_2_. P1, indicated by the blue underlining, contains the predicted ArgR_2_ binding box 1 (ArgR_2_-1). P2, indicated by the red underlining, contains the predicted ArgR_2_ binding box 2 (ArgR_2_-2). P3, indicated by the green underlining, contains the predicted ArgR_2_ binding box 3 (ArgR_2_-3). (C) EMSAs of His-ArgR_2_ with biotin-labeled P1, P2, and P3. S represents the negative control using the unlabeled probe.

ArgR was involved in the metabolism of l-arginine, which usually bound to a 16- to 20-bp conserved palindrome sequence (named the ARG box) in the promoter of the arginine biosynthetic genes. ArgR inhibited the expression of these genes, thereby regulating the arginine concentration. As shown in Fig. 6A, the ARG box is composed of 18 bases, and the AT content is relatively high (https://regprecise.lbl.gov/sites.jsp?regulog_id=4344). According to the conserved motif, two typical ARG boxes, ArgR_2_ box 2 (ArgR_2_-2) and ArgR_2_-3, were identified within the regulatory region of the *cla* operon in addition to ArgR_2_-1 predicted by Softberry (Fig. 6B).

To identify the precise binding site of ArgR_2_, EMSAs were performed using purified His-ArgR_2_ with three biotin-labeled probes (P1, P2, and P3) containing the predicted ArgR_2_ binding boxes separately (Fig. 6B). As shown in Fig. 6C, all three biotin-labeled probes contributed to binding to His-ArgR_2_, and P1 possessed the strongest binding sites. To further verify the potential binding sequence, the key sites were mutated. The conserved T was mutated to C, and A was mutated to G. The results showed that the mutated probe still bound to ArgR_2_ (data not shown). However, under the same conditions, the mutational probes P1* and P3* showed a reduction in binding, while mutation of the second site showed no significant difference, suggesting that the ArgR-1 and ArgR-3 boxes might be the main binding sites of ArgR_2_.

## DISCUSSION

**FIG 7:**
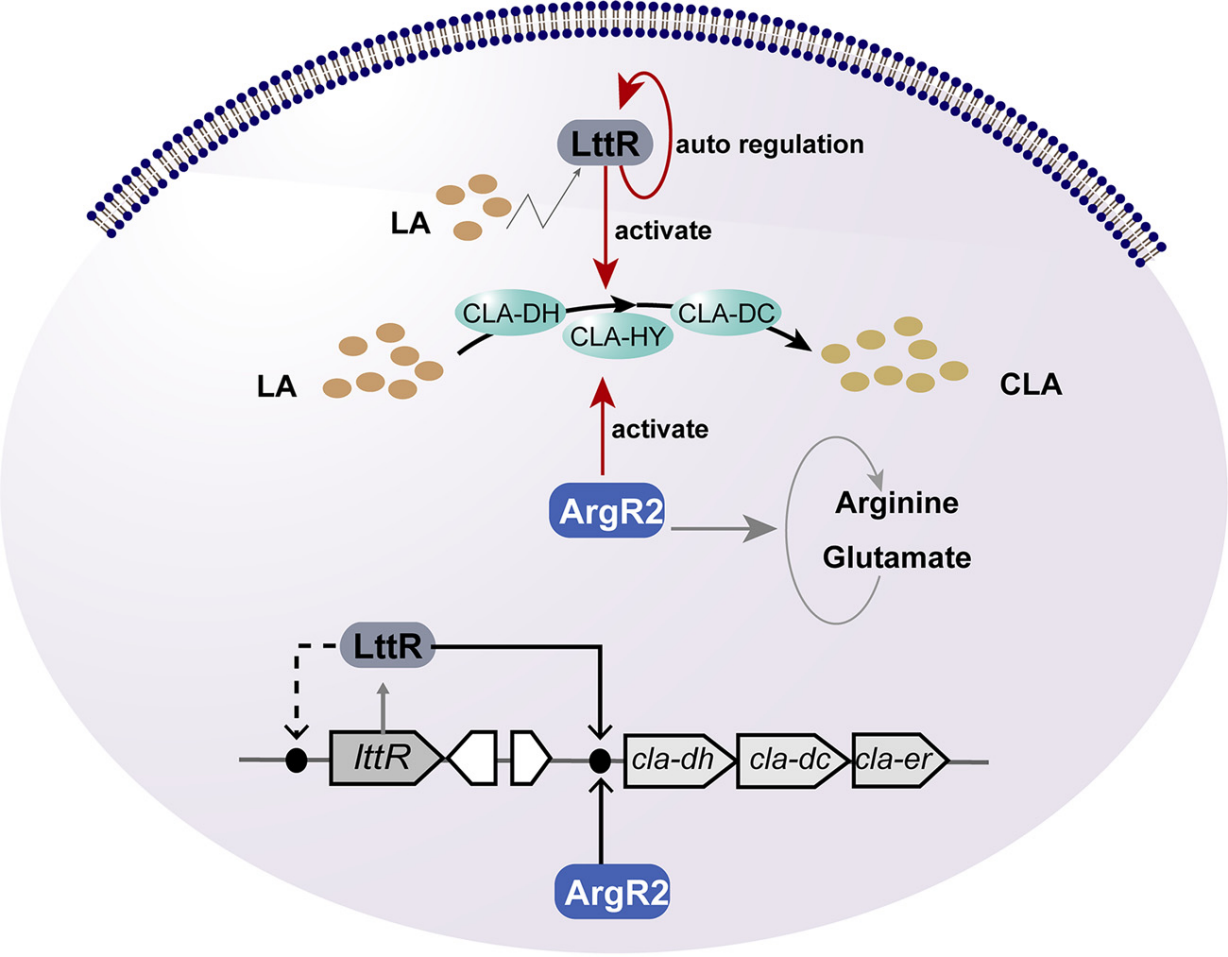
The transcriptional regulatory mechanisms of CLA biosynthesis in *L. plantarum*.

CLA has gained much attention due to its important physiological functions. But little is known about its biosynthesis and regulation mechanisms. In this study, we found that the arginine repressor ArgR_2_ bound to the promoter of the *cla* operon and regulated the biosynthesis of CLA in *L. plantarum* AR195. The deletion of *argR_2_* inhibited the transcription of *cla-dh* and *cla-dc*, thereby reducing the CLA yield. Through *cis*-element analysis and segmental EMSAs, three AT-rich ArgR_2_ binding boxes were identified. Among them, the ArgR-1 and ArgR-3 boxes played a major role in this regulation. Our previous work showed that LttR positively regulates CLA production in *L. plantarum* WCFS1. Taking this one step further, the current study focused on *L. plantarum* AR195 (the high-CLA-producing strain screened in our laboratory bank) and found that in addition to LttR, ArgR_2_ also positively regulated the transcription of *cla-dh* and *cla-dc*. This work demonstrated that ArgR_2_ mediated the interplay between fatty acid metabolism and amino acid metabolism and suggested that there were fine regulatory mechanisms during CLA biosynthesis (Fig. 7).

ArgR is a hexamer protein that can inhibit the transcription of arginine biosynthesis-related genes. ArgR in Escherichia coli has been studied thoroughly: an “SR” sequence in the N-terminal domain participates in DNA binding, and a conserved sequence, “GTIAGDDTL/I,” at the C terminus is considered to be the arginine binding domain (21–23). The binding of ArgR and ArgR boxes in the DNA regulatory region caused topological and structural changes in genes, thus regulating the transcription of target genes. In addition to regulating arginine synthesis, ArgR is involved in the regulation of nitrogen metabolism, purine and pyrimidine biosynthesis, cell morphology, and antibiotic biosynthesis in Streptomyces coelicolor (24). In this study, ArgR_2_ of *L. plantarum* acted as a transcriptional activator promoting CLA biosynthesis, suggesting possible cross talk between arginine and fatty acid metabolisms mediated by ArgR_2_ (Fig. 7).

This study furthers our understanding of CLA biotransformation in *L. plantarum* and broadens our knowledge of ArgR. It also lays a theoretical foundation for the biosynthesis and regulation of CLA.

## MATERIALS AND METHODS

### Strains, plasmids, and growth conditions.

The strains and plasmids used in this study are listed in Table 1. E. coli and its derived strains were cultured in Luria-Bertani (LB) medium at 37°C at 200 rpm. The seed activation of *L. plantarum* AR195 and the derived strains were cultured at 37°C on MRS medium agar plates under anaerobic conditions. The activated colonies were inoculated into MRS liquid medium containing LA for CLA biotransformation as described previously (20).

**TABLE 1:** Strains and plasmids used in this study

Strain or plasmid	Description	Source or reference
Strains		
* *E. coli BL21(DE3)	Protein expression host	Novagen
* L. plantarum* AR195	Wild-type strain	19
* L. plantarum* AR195 Δ*argR_2_*	*argR_2_* knockout strain	This study
* L. plantarum* AR195/pIB184*-argR_2_*	*argR_2_* overexpression strain	This study

Plasmids		
pET30a	Protein expression vector	Novagen
pET30a-*argR_2_*	ArgR_2_ expression recombinant vector	This study
pLCNICK-0537	Knockout plasmid	26
pLCNICK*-argR_2_*	Used for *argR_2_* knockout	This study
pIB184	Overexpression plasmid	20
pIB184*-argR_2_*	Used for *argR_2_* overexpression	This study

### Genome sequencing.

The genome of *L. plantarum* AR195 was sequenced using high-throughput sequencing technology. The genome sequence was assembled from scratch by means of bioinformatics. The complete genomic sequence was sequenced by the second- and third-generation Illumina HiSeq and PacBio platforms. Genome sequencing and analysis were performed by Shanghai Majorbio Bio-pharm Technology Co. Ltd. (Shanghai, China).

### DNA pulldown.

The promoter regions of the *cla* operon of *L. plantarum* AR195 were amplified by PCR using the primer pair qd-0080-A/S listed in Table 2. The PCR products were labeled with biotin using a universal biotin-labeled primer (5′-biotin-AGCCAGTGGCGATAAG-3′). The biotin-labeled DNAs were bound to streptavidin beads for affinity chromatography. The *L. plantarum* cells were harvested by centrifugation and resuspended in phosphate-buffered saline (PBS) buffer after washing. The components after ultrasonic crushing were centrifuged at 5,000 × *g* for 30 min. The supernatant was incubated with the beads, which bound to DNA. After nonspecifically bound proteins were eluted, the retained proteins were eluted with different concentrations of eluates. Twenty microliters of eluted proteins was used for SDS-PAGE, and the bands were cut off for liquid chromatography-tandem mass spectrometry (LC-MS/MS) analysis (Jingjie PTM BioLab Co. Ltd., Hangzhou, China). Proteins were matched against the NCBInr and Swiss-Prot/UniProt databases.

**TABLE 2:** Primers used in this study

Primer	Sequence
pET30a-argR_2_-S	5′-CGGGGTACCGTGAAGAAGCAAGAGCGCCA-3′
pET30a-argR_2_-A	5′-CGGAATTCGTGATCACTCAGTAAGCGTTGAAT-3′
PET-YZ-S	5′-CATCATTCTTCTGGTCTGGTG-3′
PET-YZ-A	5′-ACCCCTCAAGACCCGTTTAG-3′
PIB184-argR_2_-S	5′-ATGACAATGATGTTGGATCCGTGAAGAAGCAAGAGCGC-3′
PIB184-argR_2_-A	5′-GCTTATCGATAGATCTCGAGTTAGTGATCACTCAGTAAGCGT-3′
PIB-YZ-S	5′-GAGGAAGCGGAAGAGCGTCT-3′
PIB-YZ-A	5′-GCAGTGAGAGCGAAGCGAAC-3′
argR_2_-up-S	5′-CTTTTTCTAAACTAGGGCCCCAAACGGCGTTAGTAAAAGCTAGTG-3′
argR_2_-up-A	5′-GGTCGAAGATAGCTTACTAACCGATCACCCCCGGTAGC-3′
argR_2_-down-S	5′-TAGCTACCGGGGGTGATCGGTTAGTAAGCTATCTTCGACCTGC-3′
argR_2_-down-A	5′-ACCGAGTCGGTGCTTTTTTTCCTCGTCGAATTCTTGCA-3′
argR_2_-sgRNA-S	5′-TTTGCAAGAATTCGACGAGGAAAAAAAGCACCGACTCG-3′
argR_2_-sgRNA-A	5′-ATACTATGATATATTCTAGACTATGTCTCGTATGCGACTCGTTTTAGAGCTAGAAATAGCAAGT-3′
PLCP-YZ-S	5′-AAGGGATAGTAATTCATTCCTG-3′
PLCP-YZ-A	5′-AGGTTCTTATGGCTCTTGTATC-3′
argR_2_-YZ-S	5′-CACTACGCTGTCCAGGCAG-3′
argR_2_-YZ-A	5′-TCGCCCGTCGTAAGTAGTTG-3′
qd-0080-S	5′-AGCCAGTGGCGATAAGGCACAATGAACGGTTGCAGTAT-3′
qd-0080-A	5′-AGCCAGTGGCGATAAGGATGTTTTGCGTCCTCCTCG-3′
Bio-EMSA	5′-biotin-AGCCAGTGGCGATAAG-3′
0080qdz-1-S	5′-AGCCAGTGGCGATAAGTGACAATGAACGGTTGC-3′
0080qdz-1-A	5′-AGCCAGTGGCGATAAGAGTGTGGTTCCAACTTTAAT-3′
0080qdz-2-S	5′-AGCCAGTGGCGATAAGTTAGTTAGTTATAATTGTGAACTAAC-3′
0080qdz-2-A	5′-AGCCAGTGGCGATAAGGACTACGCTTTCATATTAACC-3′
0080qdz-3-S	5′-AGCCAGTGGCGATAAGAAGATAAATGACCGGTCATCA-3′
0080qdz-3-A	5′-AGCCAGTGGCGATAAGGATGTTTTGCGTCCTCCTC-3′
0080qPCR-S	5′-AAGCGACCGCCTATGAC-3′
0080qPCR-A	5′-GGTTTCCCGTTGGTAATG-3′
0081qPCR-S	5′-CTCGGTGCCGCTTGGTT-3′
0081qPCR-A	5′-TCGCTGCCTTGGGATTG-3′
0082qPCR-S	5′-GATTGATTGTTCCGTTGTC-3′
0082qPCR-A	5′-CGTATGGAGCGGTTCTT-3′
16sRNA-S	CAAGGCTGAAACTCAAAGGA
16sRNA-A	GACGACAACCATGCACCAC

### Heterologous expression and protein purification.

The *argR_2_* genes were amplified from *L. plantarum* AR195 genomic DNA by PCR using primers (pET30a-argR_2_-S/A) listed in Table 2. The purified *argR_2_* sequences were inserted into pET30a digested by EcoRI and KpnI, generating the recombinant vector. For ArgR_2_ expression, the recombinant plasmids were extracted from the transformant and verified by PCR using the primer pair PET-YZ-S/A. The positive plasmids verified by PCR and sequencing were introduced into the expression host, E. coli BL21(DE3). Colony PCR was performed using a 2× colony PCR mixture, and the products were detected by agarose gel electrophoresis. Protein expression and purification were performed as described previously (20).

### EMSA.

The biotin-labeled *cla* regulatory regions were amplified by two-step PCRs, the same as that described above for the DNA pulldown assay. The quality and concentration of the probes were determined by agarose gel electrophoresis and by using a Nanodrop 2000 spectrophotometer (Thermo Fisher Scientific). EMSAs were carried out using a chemiluminescent EMSA kit (Beyotime Biotechnology, China) as described previously (25).

### Octet.

The preparation of biotin-labeled probes used for Octet analysis was the same as that used for EMSAs. The biotin-labeled probes were dissolved in buffer A containing 10 mmol/L HEPES, 2 mmol/L MgCl_2_, 0.1 mmol/L EDTA, and 200 mmol/L KCl (pH 8.0). Different concentrations of ArgR_2_ were dissolved in buffer B containing 1‰ (wt/vol) bovine serum albumin (BSA) and 2‰ (vol/vol) Tween 20; the other components were the same as those in buffer A. The samples were added to the detection plate and determined by Octet analysis based on the theory of biolayer interferometry (BLI). The determination procedure was as follows: balancing in buffer A for 10 min, loading of DNA probes for 10 min, balancing in buffer B until the baseline was flat, association with protein for 10 min, and dissociation in buffer B for 10 min (25).

### Mutant strain construction.

The *argR_2_* deletion strain of *L. plantarum* was constructed using CRISPR-Cas9 gene-editing technology. The upstream and downstream homologous arms of *argR_2_* and the single guide RNA (sgRNA) were inserted into pLCNICK-0537, which was digested by XbaI and ApaI (Thermo Fisher Scientific, USA) (26). The positive recombinant knockout plasmid pLCNICK-*argR_2_* was verified by colony PCR and sequencing. The upstream verification primers were designed at the upstream 249 bp of the upper homologous arm, and the downstream primers were designed at the downstream 199 bp of the lower homologous arm. The expected PCR product length of wild bacteria is 2,907 bp. If *argR_2_* is successfully knocked out, the length should be 2,448 bp. The positive recombinant plasmids were introduced into *L. plantarum* competent cells (20).

### CLA determination.

CLA has a special absorption peak at 233 nm, while linoleic acid does not. According to the optical properties of CLA, a UV spectrophotometer was used to determine the CLA concentration. Different concentrations of a CLA standard were dissolved in *n*-hexane, and a standard curve was drawn according to the absorbance at 233 nm. Two milliliters of isopropanol and 1.5 mL of *n*-hexane were added to 1 mL of fermentation broth and centrifuged after vortex oscillation. The supernatant was removed and washed with distilled water. After centrifugation, the supernatant was dried with anhydrous sodium sulfate. Next, it was dissolved in 5 mL *n*-hexane for UV absorption detection. The concentration of CLA was calculated according to the standard curve.

### qPCR.

The primers used for quantitative PCR (qPCR) are listed in Table 2. The strains were harvested for RNA purification at the exponential phase. The WT strains were sampled at the 6th hour, and the mutant bacteria were sampled at the 12th hour. Cell culture and RNA extraction were performed using a total RNA extraction kit (Tiangen Biotech, Beijing, China) according to the manufacturer’s instructions. DNase digestion was introduced to remove the genomic DNA. The cDNA was prepared and analyzed by real-time fluorescence quantitative PCR as previously described (19, 20). The fold changes in mRNA were calculated by the 2^−ΔΔ^*^CT^* method.

### Data availability.

The FASTQ format raw data have been deposited in the National Center for Biotechnology Information Sequence Read Archive (NCBI SRA) database (accession no. PRJNA765134).
